# M7G-related tumor immunity: novel insights of RNA modification and potential therapeutic targets

**DOI:** 10.7150/ijbs.90382

**Published:** 2024-01-27

**Authors:** Mengzhen Han, Qibo Huang, Xinxin Li, Xiaoping Chen, He Zhu, Yonglong Pan, Bixiang Zhang

**Affiliations:** 1 Hepatic Surgery Center, Tongji Hospital, Tongji Medical College, Huazhong University of Science and Technology, Wuhan, Hubei 430030, China.; 2 Hubei Key Laboratory of Hepato-Pancreato-Biliary Diseases, Wuhan, Hubei 430030, China.

**Keywords:** N7-methylguanosine (m7G), RNA modification, Tumor immunity, Adjuvant therapy, Biomarker

## Abstract

RNA modifications play a pivotal role in regulating cellular biology by exerting influence over distribution features and molecular functions at the post-transcriptional level. Among these modifications, N7-methylguanosine (m7G) stands out as one of the most prevalent. Over recent years, significant attention has been directed towards understanding the implications of m7G modification. This modification is present in diverse RNA molecules, including transfer RNAs, messenger RNAs, ribosomal RNAs, and other noncoding RNAs. Its regulation occurs through a series of specific methyltransferases and m7G-binding proteins. Notably, m7G modification has been implicated in various diseases, prominently across multiple cancer types. Earlier studies have elucidated the significance of m7G modification in the context of immune biology regulation within the tumor microenvironment. This comprehensive review culminates in a synthesis of findings related to the modulation of immune cells infiltration, encompassing T cells, B cells, and various innate immune cells, all orchestrated by m7G modification. Furthermore, the interplay between m7G modification and its regulatory proteins can profoundly affect the efficacy of diverse adjuvant therapeutics, thereby potentially serving as a pivotal biomarker and therapeutic target for combinatory interventions in diverse cancer types.

## Introduction

The progression of detection technologies in recent decades has led to a heightened emphasis on investigating the biological roles of RNA modifications 1. RNA modifications are chemical alterations that take place within the ribose and nucleobase constituents. These alterations significantly impact the structural conformation and functional properties of RNA molecules 2-4. There are more than 150 types of identified RNA modifications, including pseudouridine (ψ), N6-methyladenosine (m6A), N1-methyladenosine (m1A), 5-methylcytosine (m5C), N7-methylguanosine (m7G), N4 -acetylcytosine (ac4C), uridylation, adenosine-to-inosine (A-to-I) RNA editing, etc 5,6. Among these different RNA modifications, m6A is most commonly found in messenger RNA (mRNA) modification, m5C in transfer RNA (tRNA) can influence its stability and translational fidelity, while ψ mainly present in non-coding RNA (ncRNA) like ribosomal RNA (rRNA) and small nuclear RNAs (snRNAs) 7-9. More recently, researchers also reported several RNA modifications in microRNAs (miRNAs), long noncoding RNAs (lncRNAs) and small nucleolar RNAs (snoRNAs) 10,11. Previous studies have focused on the m6A modification and its role in diverse biological processes 12. However, further research is necessary to explore additional non-m6A chemical modifications in various types of RNAs.

M7G modification is a methylation of the seventh N on RNA guanylate which can introduce positively charged or amphiphilic ions into nucleobases 13,14. The m7G modification can be categorized into two distinct types based on the position of the methylated guanylate: m7G-caps modification and internal m7G modification. Initially, the modification of m7G-caps was detected on the 5′ caps of eukaryotic mRNAs. These m7G-caps play a pivotal role in shielding mRNA from degradation while facilitating essential caps-associated mRNA processes, such as splicing, nuclear export, and translation 15,16. Furthermore, m7G methylation of internal positions was demonstrated in mRNAs, tRNAs and rRNAs, which could also influence the distribution, structure and function of them 17,18. With the current advancements in methods for detecting m7G-methylated RNAs and high-throughput technologies, an increasing number of methylation sites and diverse types of non-coding RNAs (ncRNAs) have been found to be associated with m7G modification 19,20. Moreover, earlier studies have proposed that disruption in m7G methylation may link to a spectrum of ailments, encompassing cardiovascular diseases, neurological disorders, and various types of cancers 13,15,21.

Cancer stands as a significant public health challenge and represents the second leading cause of global mortality. The reprograming of immune system and evasion of cancer cells from immune destruction are important hallmarks of cancer, which have been sufficiently validated in past decades 22,23. Recent researches demonstrated that m7G methylation could regulate immune cells activation and infiltration, which were related to immune surveillance and immune evasion 13,24. Furthermore, there is a growing focus on tumor-targeted therapies, particularly within the realm of cancer immunotherapy. Notably, the influence of m7G methylation extends to the efficacy of such therapeutic interventions 5. This comprehensive review primarily aims to elucidate the role of RNA m7G modification in the context of tumor immunity and targeted therapeutic approaches. The insights garnered herein hold the potential to furnish promising strategies for advancing cancer therapy.

## M7G regulators and their roles in cancers

**Figure 1:**
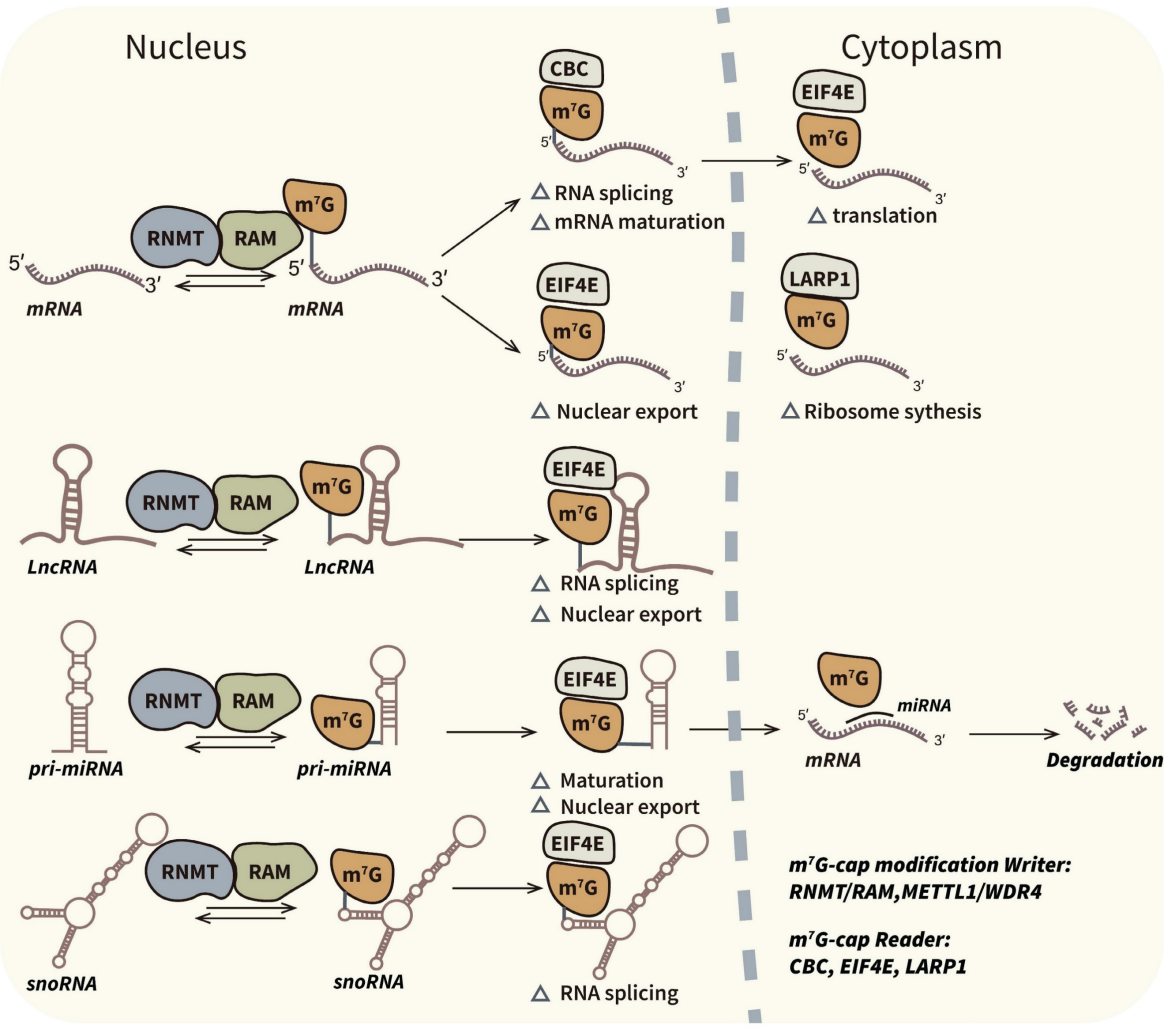
** Biological mechanism and associated regulators of m7G-cap modification.** M7G-cap modification occur in 5′ terminal guanosine of mRNAs and some ncRNAs such as miRNAs, lncRNAs and snoRNAs. M7G capping is an important post-transcriptional process which can influence transcripts splicing, maturation, nuclear export and translation. RNMT/RAM complex is the confirmed writer in m7G-cap modification. CBC, EIF4E and LARP1 are readers which can bind to the m7G caps.

**Figure 2:**
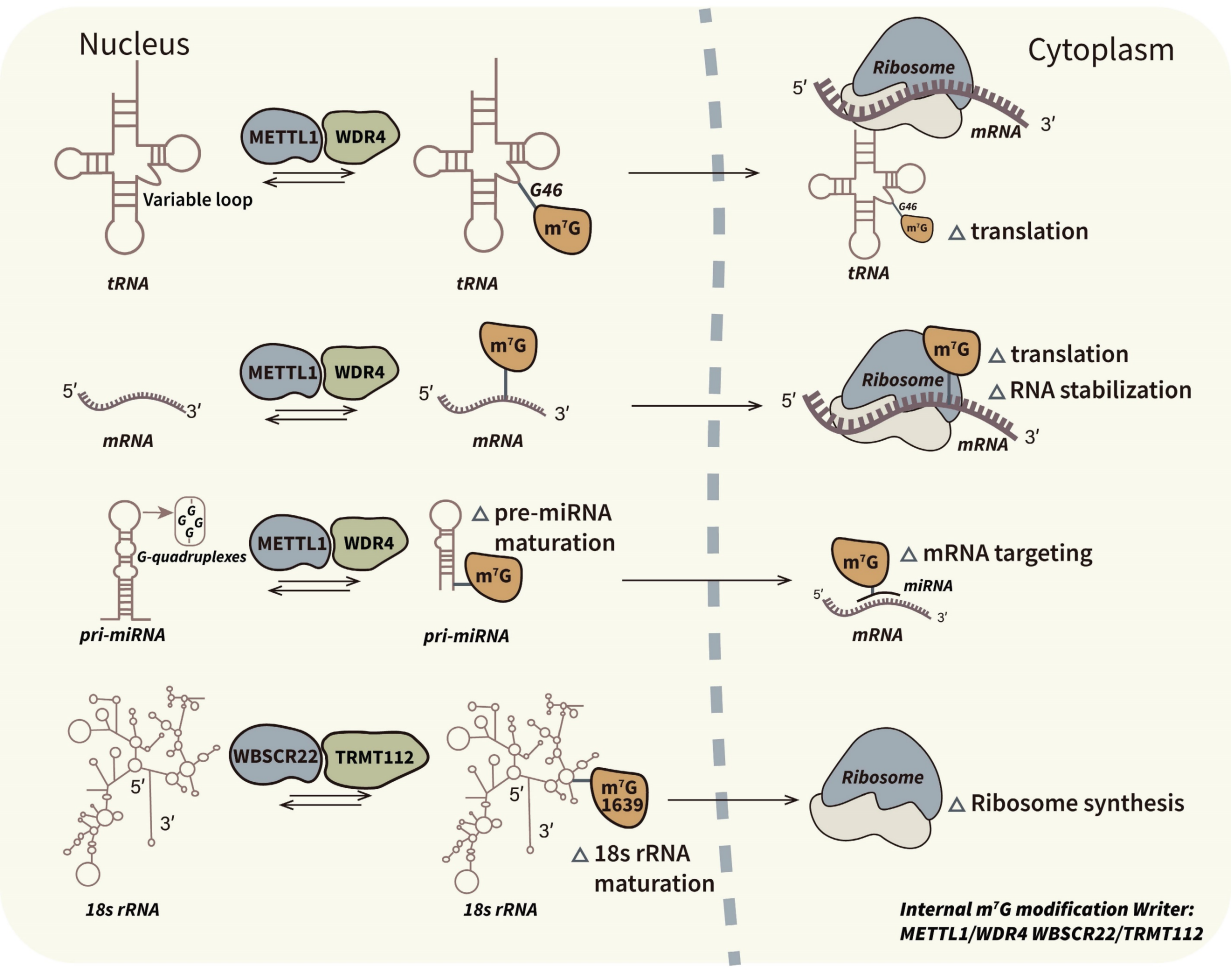
** Biological mechanism and associated regulators of internal m7G modification.** M7G modification has been demonstrated at internal positions of different types of transcripts. Internal m7G modification plays pivotal roles in RNA stabilization, translation initiation and ribosome synthesis. METTL1/WDR4 complex is the most studied internal m7G writer which catalyze methylation of tRNAs, mRNAs and pri-miRNAs. And WBSCR22/TRMT112 complex is the specific m7G writer of rRNAs.

Eukaryotic RNA methylation is typically governed by three distinct categories of proteins: methyltransferases (referred to as "writers"), methylation binding proteins (commonly known as "readers"), and demethylases (known as "erasers") 25,26. Previous research has substantiated the presence of various regulators associated with m7G modification. In yeast, it has been reported that Abd1 functions as a writer responsible for m7G capping of mRNA at the 5' end, while the Trm8/Trm82 complex is responsible for internal m7G modification of tRNAs 18,27. In humans, regulators of m7G modification encompass a writer complex that includes RNMT/RAM, METTL1/WDR4, and WBSCR22/TRMT112, along with the reader complexes cap-binding complex (CBC) and eukaryotic translation initiation factor 4E (EIF4E) that recognize m7G caps 28,29. Furthermore, recent investigations have made significant strides in identifying internal m7G binding proteins, which may serve as novel readers of m7G modifications. This paper focuses on elucidating the functions of these regulators in the context of cancer. Figure 1 and 2 depict the relevant regulators and mechanisms associated with m7G capping and internal m7G modification.

### Writers

#### RNMT/RAM

M7G-cap modification is a tightly regulated process occurred in 5′ terminal guanosine of mRNAs and certain ncRNAs, which is required for transcripts splicing, cleavage and polyadenylation (CPA), nuclear export and translation initiation in eukaryotes 30. In the beginning, RNA guanine-7 methyltransferase (RNMT) was considered to be the monomer methyltransferase to catalyze 5' cap methylation by utilizing methyl of S-adenosyl methionine (SAM) 31. Nevertheless, more recent researches demonstrated that RNMT-Activating Miniprotein (RAM) could bind to RNMT and form an enzyme complex, thereby stabilize and allosterically activate RNMT, as well as increase its affinity for RNA 31-33. The RNMT/RAM complex is mainly located in nucleus, and the RNAs can directly bind to m7G-cap readers shortly after capping process 34.

Function of RNMT/RAM complex in m7G-cap modification could be regulated by distinct molecules and signaling pathways, which is also associated with various cancers. For example, recent publications suggested that the classical tumorigenic molecule c-MYC could upregulate the m7G-capping process catalyzed by RNMT. In breast cancers, c-MYC was reported to combine with RNMT and enhance the translation of cyclin D1 through the induction of m7G-capping, thus facilitate the malignant transforming of mammary epithelial cells 35. Similarly, a model of Burkitt's lymphoma showed that c-MYC could recruit RNMT to promoters of WNT pathway genes and promote m7G-capping of these transcripts 36. Additionally, CDK1 and cyclin B1 could phosphorylate RNMT at Thr77, consequently elevate m7G-cap modification levels and induce cell proliferation 37. The m7G-cap reader EIF4E were also found in regulation of RNMT, which could serve as co-factor and facilitate the capture of capped RNAs 38.

#### METTL1/WDR4

The methyltransferase-like 1 (METTL1) and WD repeat domain 4 (WDR4) complex was firstly reported to act as the writer of m7G modification at tRNA variable loop G46^39^. Within this complex, METTL1 recognizes tRNA via its αC and α6 helices, inducing tRNA bending through its N-terminal regions (residues 1-33) while performing catalytic functions. Simultaneously, WDR4 interacts with METTL1, serving as a scaffold to facilitate tRNA binding 40,41. Earlier investigations have proposed that METTL1 can undergo phosphorylation at Ser27 by AKT and RSK, leading to reduced methyltransferase activity^42^. The m7G modification by the METTL1/WDR4 complex stabilizes tRNA structures, prevents frame shifting, and enhances the translation of mRNAs enriched with m7G-decoded codons 43. Dysregulation of tRNA m7G modification by METTL1/WDR4 complex and consequent codon-biased translation may contribute to various types of cancers 44,45. For instance, studies in lung cancer have illustrated that overexpression of METTL1 and WDR4 stabilizes m7G-modified tRNA ValAAC and ProAGG, consequently promoting the translation of genes involved in cell-cycle regulation, thereby influencing tumor progression and prognosis 46. Similar upregulations of oncogenic translation were also indicated in hepatocellular carcinoma (HCC) and intrahepatic cholangiocarcinoma (ICC) 47,48. In esophageal squamous cell carcinoma (ESCC), researchers demonstrated that METTL1 and WDR4 could induce the translation of RPTOR and decrease autophagic flux in ESCC cells 49. Apart from these types of solid tumors, METTL1/WDR4 complex was reported to be involved in oncogenic transformations of acute myelocytic leukemia (AML) in an tRNA m7G dependent manner 50. These results showed important function of tRNA m7G modification regulated by METTL1/WDR4 complex in cancers.

**Figure 3:**
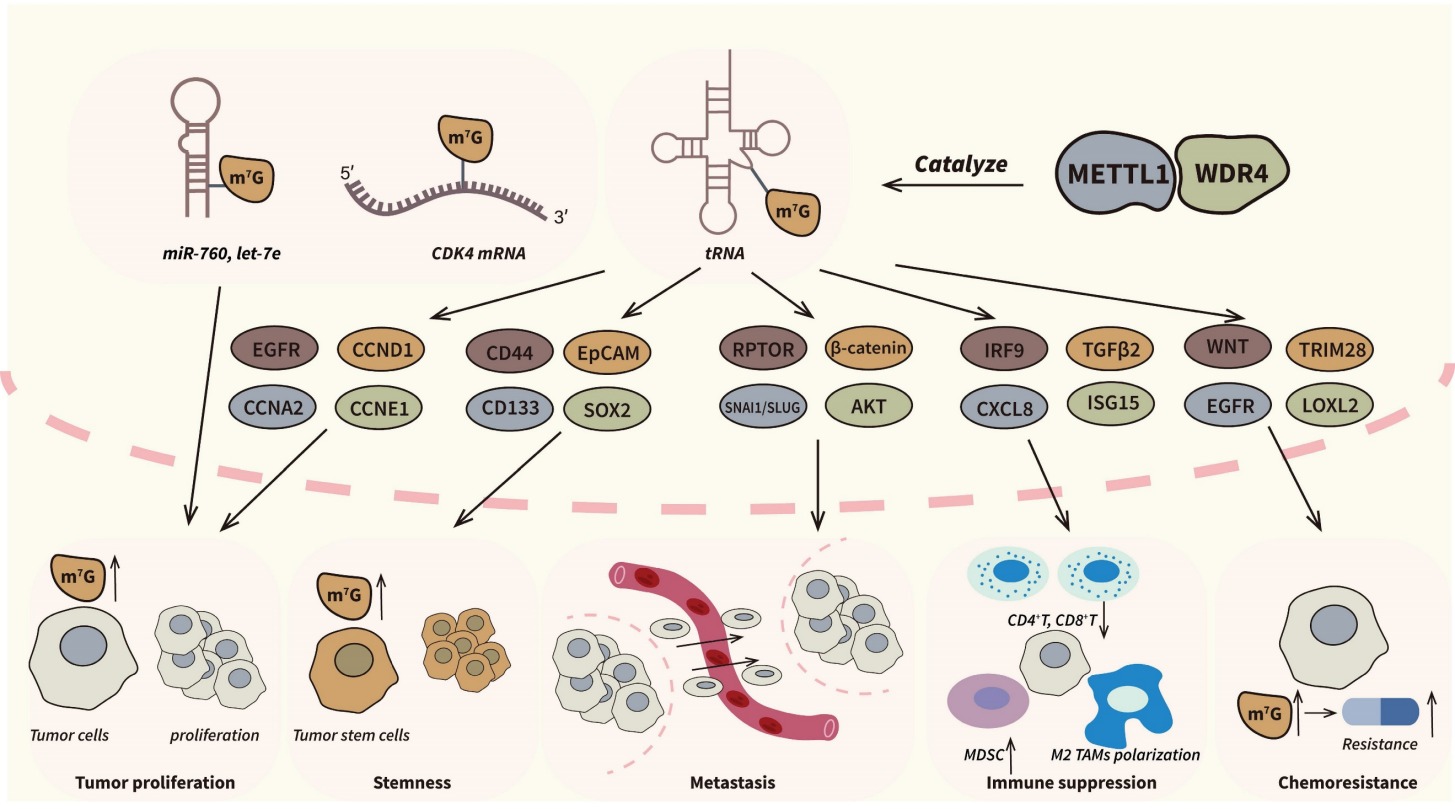
** The roles of METTL1/WDR4 complex in cancers.** METTL1/WDR4 complex is the most extensively researched m7G writer and an important oncogenic factor in many types of cancers. The complex can regulate a series of pathological processes in tumor progression including proliferation, metastasis, stemness, immune suppression and chemoresistance by m7G dependent manners. The dysregulated pathways and genes are showed above.

Except for tRNA, METTL1/WDR4 can also catalyze m7G modification of mRNA and miRNA 51. Transcriptome-wide profiling has unveiled the ability of the METTL1/WDR4 complex to introduce m7G modifications into mRNA molecules, conferring protection against degradation and enhancing their translational efficiency 19. As internal m7G modification of mRNAs mainly occur in GA-rich context, researchers further indicated that METTL1/WDR4 complex could bind to GA-rich motifs and exert its methyltransferase activity 17,52. Notably, in prostate cancer, METTL1 stabilizes CDK14 mRNA by catalyzing internal m7G modification, resulting in the upregulation of CDK14, which in turn promotes tumor cell proliferation and invasion 53. Additionally, Pandolfini et al. firstly reported the m7G modification of miRNA let-7. Their results indicated that METTL1 could facilitates disruption of the secondary structure G-quadruplexes and promote pre-miRNA maturation 54. Similarly, researches in bladder cancer showed that METTL1 could accelerate tumor progression by overexpressing miR-760 in an m7G-dependent manner 55. Furthermore, recent studies also reflected the potential m7G modification in lncRNA, which further illustrate the biological function of METTL1/WDR4 complex 56. A summary of the functions of the METTL1/WDR4 complex in cancers is provided in Figure 3.

#### WBSCR22/TRMT112

The Williams-Beuren syndrome chromosomal region 22 (WBSCR22), also known as metastasis-related methyltransferase 1 (MERM1), was initially identified as an essential pro-metastatic regulator involved in breast cancer metastasis 57. WBSCR22 functions as a functional homolog of the yeast Bud23 methyltransferase, possessing an SAM binding domain that facilitates the catalysis of 18S rRNA m7G modification at position G1639, thus expediting its maturation 58,59. These processes play a crucial role in the biogenesis of 40S ribosomal subunits 60. WBSCR22 has the capacity to interact with its co-factor and activator, the tRNA methyltransferase activator subunit 11-2 (TRMT112), forming a heterodimeric methyltransferase complex responsible for rRNA m7G modification 61. Previous studies have demonstrated that TRMT112 can stabilize WBSCR22 by inhibiting its Ubiquitin-Proteasome degradation 64. WBSCR22 and TRMT112 have been implicated in tumor progression and chemoresistance across various cancers. For instance, recent publications in colorectal cancers (CRC) have suggested that the overexpression of WBSCR22 correlates with decreased overall survival and oxaliplatin resistance 62. Conversely, researchers have also elucidated the tumor-suppressive functions of WBSCR22 and TRMT112 in pancreatic cancers 63. Nevertheless, the specific mechanisms underlying rRNA m7G modification by the WBSCR22/TRMT112 complex remain underexplored in the context of cancer, and further investigation is warranted to explore other potential m7G writers.

### Readers and erasers

In the realm of RNA methylation, erasers possess the ability to reversibly remove methyl groups from RNA, while readers encompass binding proteins that can recognize specific methylation sites and facilitate the actions of both writers and erasers 4. Currently, the body of research pertaining to m7G modification remains insufficient, and there exists no comprehensive report on m7G erasers. CBC and EIF4E are binding factors identified as m7G-caps readers, involving in various posttranscriptional processes of RNAs. CBC consists of the m7G-cap binding protein NCBP2 and the homologue NCBP1 64. Following m7G-caps modification by RNMT, mRNAs engage with NCBP2 and undergo a series of maturation steps, including splicing, CPA, and nuclear export 65. Subsequently, cytoplasmic EIF4E assembles on the m7G-caps and recruits mRNAs to the translation machinery 66. Notably, EIF4E is not limited to mRNAs; it can also engage in m7G-caps modification of certain ncRNAs, such as lncRNAs, miRNAs, and snoRNAs 67,68. Among them, nuclear EIF4E can recognize m7G-caps of lncRNAs and promote their export, and m7G-capped pre-miRNAs are more likely to generate single 3p-miRNAs because of the biased selection by Ago. EIF4E is a prooncogenic molecule overexpressed and involved in many types of cancers. In AML, nuclear export of m7G-cap modification mRNA by EIF4E induce translation of c-MYC and Mcl-1, which are associated with relapse and poor prognosis 69. Likewise, recent studies in osteosarcoma (OS) cells showed that EIF4E could enhance translation of cyclin D1 as well as mTOR pathway genes 70,71. certain other family members of EIF4E can also exert critical roles in various cancer types through m7G capping, including the tumor suppressor EIF4E3 72.

More recently, potential internal mRNA m7G-binding proteins have been identified via RNA pull-down assays. The results suggested that Quaking proteins (QKIs) could recognize and interact with m7G-modified oligonucleotides, then regulate stability and translation efficacy of mRNA under stress conditions. subsequently regulating the stability and translation efficiency of mRNA under stress conditions. In cancer cells, QKI7 has demonstrated the ability to mitigate chemoresistance to doxorubicin and sorafenib by reducing the translation of certain associated genes, including ROCK1, GSK3B, TEAD1, and IGF1R 52. As the development of MeRIP-seq progresses, it is anticipated that more internal m7G readers will be identified, potentially shedding additional light on the mechanisms of m7G modification and its roles in cancer.

### Other regulation factors in m7G modification

In addition to the determined m7G writers and readers discussed earlier, various internal and external factors play a pivotal role in influencing m7G processes and the expression of m7G regulators. These regulatory factors are commonly recognized as upstream mechanisms of m7G modification with significant implications in tumor initiation and progression. Internal factors involved in the regulation of m7G modification encompass oncogenic molecules or pathways, along with epigenetic modifications. Prior investigations have suggested that the classical m7G writer METTL1 may undergo transcriptional upregulation by the transcription factor (TF) SP1, thereby stabilizes CDK14 mRNA and contributes to the progression of prostate cancers 53. Whole exon sequencing in nasopharyngeal cancer (NPC) has elucidated the downregulation of METTL1 by the pivotal tumor suppressor ARNT 73. Similarly, the transcription of WDR4 was noted to be activated by c-MYC, thereby potentially fostering HCC progression 74. Furthermore, the analyses of m7G genetic variation revealed that the cope number variation (CNV) and single nucleotide variation (SNV) of m7G regulators were prevalent in various cancers, and researchers also demonstrated the correlation between the methylation level and expression of m7G regulators 75. Regarding the external factors, m7G modification processes can be triggered under stress conditions, such as ionizing radiation (IR), heat shock and drug treatments. Recent studies have indicated that chemotherapeutic treatments in diverse tumor cells can induce mRNA m7G modification mediated by METTL1 and QKI7. Furthermore, radiotherapy in HCC has been associated with the overexpression of METTL1^52^,^76^. Both internal and external regulatory factors predominantly influence m7G regulators, and these investigations hold promise for devising effective strategies for combination therapies across various cancer types.

## The roles of m7G modification in immune cell biology

Immune system can exert its function of immune surveillance and immune response against tumor cells, which play crucial roles in proliferation, metastasis and treatment resistance of cancers 77,78. Tumor immune microenvironment (TIME) consists of tumor, stromal and immune cells including adaptive and innate immune cells 79. Current studies have showed remarkable enrichment of m7G regulators in biological processes in TIME, especially immune infiltration 6. Here, we reviewed the studies of prognostic models based on m7G levels and concluded activation or accumulation of immune cells mediated by m7G modification. The detailed information is also showed in Table 1.

### T cells

T cells constitute pivotal components of the adaptive immune system, encompassing distinct subtypes such as CD4^+^ and CD8^+^αβ T cells, γδ T cells, and natural killer T (NKT) cells 80. Upon recognition of cognate antigens by T cell receptors and subsequent stimulation by cytokines, naïve CD4^+^ and CD8^+^ T cells undergo a series of intricate biological processes. These processes encompass maturation, differentiation into effector or memory cells, activation, and subsequent migration to specific anatomical locations 81,82. Prior studies have extensively explored various T cell subsets in the TIME. CD8^+^ T cells, recognized as highly responsive and effective cytotoxic T lymphocytes (CTLs), exert their antitumor effects by targeting tumor cells, predominantly through the secretion of cytotoxic proteins and the induction of cellular apoptosis 83,84. In general, the infiltration of CD8^+^ cells has been reported to display a negative correlation with m7G levels and is downregulated in high-risk patients. In the context of head and neck squamous cell carcinoma (HNSCC), cluster analysis utilizing the ESTIMATE and CIBERSORT algorithms has revealed a higher proportion of CD8^+^ T cells in the low-risk group 85. Studies conducted in CRC, prostate cancer, soft tissue sarcoma (STS), gastric cancer (GC), and ovarian cancer have suggested a similar regulatory pattern 86-90. However, CD4^+^ cells displayed varying abundances across different cancer subtypes and types. Helper T (Th) cells originate from activated CD4^+^ T cells and differentiate through distinct surface markers such as Interferon (IFN)-γ, GATA-3, and various interleukins 91-93. Among them, Th1, Th2, and Th17 cells appeared to be more stimulated in low-risk patients with GC and prostate cancer, while the analysis in STS patients indicated a reduced recruitment of Th cells 86,88,89. Regulatory T cells (Tregs) represent a subset of CD4^+^ T cells with distinctive significance in TIME. Renowned for their suppressive capabilities, Tregs exert inhibitory effects on effector T cells, thereby contributing to immune tolerance towards tumor cells 94,95. Clustering analysis performed in the context of CRC and ovarian cancers have revealed a heightened prevalence of Tregs within the high-risk patient cohort. This elevated accumulation of Tregs aligns with an associated decline in overall survival 90,96.

Numerous studies in past decades reported that m7G regulators could influence the infiltration, maturation and activation of T cells in TIME. Among these regulators, the METTL1 and WDR4 are prominently studied m7G writers within the context of T cell biology. In a comprehensive analysis of pan-cancers, overexpression of METTL1 and WDR4 was correlated with more Tregs infiltration in diverse types of cancers 97. Mechanistically, WDR4 was reported to elevate intertumoral Tregs and foster a suppressive TIME by promoting the degradation of the pleiotropic tumor suppressor promyelocytic leukemia protein and inducing CD73 expression in lung adenocarcinoma (LUAD) 98. Moreover, METTL1 has been implicated in augmenting the proportion of Tregs in HNSCC through promoting PI3K protein translation and AKT/mTOR signaling pathway activation in m7G-dependent manner 99. Additionally, the m7G-caps writer RNMT was reported to regulate CD4^+^ T cells activation. RNMT catalyzes the m7G-caps formation in genes and snoRNAs required for ribosome biogenesis, thus enhancing the translation process in activated T cells 100. WBSCR22 could also participate in activation of CD8^+^ T cells in LUAD, and the m7G-related cap-binding genes NCBP2 and EIF4E3 were suggested to involve in infiltration of CD8^+^ T cells in HNSCC 85,101. These findings indicated the role of m7G modification and its regulators in T cells biology of TIME.

### B cells

B cells perform crucial roles in adaptive immunity, encompassing antibody production, antigen recognition, and cytotoxicity 102. Tumor-infiltrating B cells can be categorized into distinct subtypes based on their stages of differentiation, including naïve B cells, activated B cells, memory B cells, and plasma cells 103. The extent of immune infiltration by these subtypes varies across different cancer types. For example, a negative correlation was observed between the infiltration of naïve B cells and the risk score for an unfavorable prognosis in CRC and LUAD. However, clustering analysis in cutaneous melanoma indicated a higher level of naïve B cell infiltration in the high-risk group 104-106.

Upon stimulation by antigens, B cells undergo activation and subsequently differentiate into two distinct cell types: memory B cells and plasma cells. Memory B cells accumulation was negatively correlated with m7G levels in GC, HCC and bladder cancer, while LUAD patients appeared opposite results 104,107-109. Plasma cells are effector cells differentiated from activated B cells which produce antibodies. Generally, tumor-infiltrating plasma cells are abundant in low-risk groups, which are also associated with better prognosis 109.

### Myeloid-derived suppressor cells (MDSCs)

MDSCs represent a population of immature myeloid cells with the capacity to suppress T-cell activity, ultimately contributing to the establishment of an immunosuppressive TIME in the advanced stages of cancer 110. MDSCs are characterized by co-expression of CD33, CD34, CD11b and IL-4Rα, and identified as two major subsets: polymorphonuclear- (PMN-) and monocytic- (M-) MDSCs 111. The classical m7G writer METTL1 was reported to regulate the accumulation of PMN-MDSCs in various cancers. In ICC, it was demonstrated that abundance of PMN-MDSCs with CD15 expression was increased in advanced tumors. Mechanistically, researchers revealed that METTL1 could promote CXCL8 translation by tRNA m7G modification, thereby activate CXCL8/CXCR2 chemokine pathways and induce the recruitment of PMN-MDSCs in TIME 112. Studies in HCC reported similar function of METTL1 in regulating PMN-MDSCs infiltration. The translation of TGF-β2 was upregulated by METTL1-dependent tRNA m7G modification, which could induce the differentiation of peripheral blood mononuclear cells (PBMCs) into PMN-MDSCs and increase their infiltration, thereby suppress the function of CD8^+^ T cells and promote HCC progression 76,113. Moreover, clustering analysis in HCC demonstrated that the m7G modification level positively correlated with abundance of MDSCs in TIME, and some novel regulators such as NUDT16 and KIF2C were involved in MDSCs regulation 114,115. On the contrary, comprehensive analysis based on m7G-related genes in skin cutaneous melanoma (SKM) suggested the higher overall survival regulated by EIF4E3 and IFIT5, which was associated with more immune infiltration including MDSCs 116.

### Macrophages

Macrophages, derived from monocytes, are integral innate immune cells 117. They hold a pivotal role within the TIME and serve as pivotal regulators across diverse biological processes including epithelial to mesenchymal transition (EMT), angiogenesis, ECM remodeling, and instigation of immunosuppressive responses 118,119. Tumor-associated macrophages (TAM) possess two polarization settings: pro-inflammatory M1 macrophages and anti-inflammatory M2 macrophages 120. Being the predominant macrophages infiltrating tumor tissues, M2 macrophages contribute to the establishment of a suppressive TIME by secreting inhibitory cytokines, inducing apoptosis in immune cells, directly attenuating TCR and BCR signaling through PD-1/PD-L1 axis, and collaborating with other immune cells to exert suppressive functions 121,122. The classical m7G writers METTL1 and WDR4 have been implicated in the polarization of macrophages towards the M2 phenotype and the establishment of a suppressive TIME. In prostate cancers, researchers found that METTL1-mediated tRNA m7G modification could impede the biogenesis of tRNA fragment, consequently diminishing the translation of transcripts associated with IFN signaling pathway and promote macrophage polarization towards the M2 phenotype 123. Likewise, overexpression WDR4 could contribute to enhanced infiltration of CD206^+^ M2 macrophages, as well as reduced CD8^+^ T cells recruitment, which induced a suppressive TIME in LUAD 98. Moreover, the clustering analysis showed a positive correlation between m7G modification level and M2 macrophages infiltration across various cancers 106,109,124,125. M1 polarization of macrophages were usually conventionally with favorable outcomes. The prognostic models of ovarian cancers, CRC, HCC and HNSCC based on m7G regulators indicated more recruitment of M1 macrophages in low-risk group of patients 85,96,115,126. However, contradictory outcomes were observed in bladder cancers 107,127. In addition, as precursor cells of macrophages, monocytes infiltration was reported to be negative correlated with m7G levels in CRC, bladder cancers and prostate cancers 89,127,128. These results are consistent with the role of monocytes in mononuclear phagocyte system.

### Neutrophils

Neutrophils serve as the primary defense mechanism during inflammatory conditions and act as effector cells to combat pathogens 129. In various types of cancers, neutrophils show the double-edged roles which can either inhibit tumor progression through cytotoxic activity or promote immunosuppression in cancers 130,131. Likewise, the influence of neutrophils infiltration by m7G related genes is still controversial. In pancreatic ductal adenocarcinoma (PDAC), researchers suggested that m7G scores based on expression of m7G regulators were positively correlated with neutrophils infiltration, and patients with high m7G scores and increased number of neutrophils appeared higher malignancy degrees and worse prognosis. PPI network showed that FN1 and ITGB1 were the core genes in regulating m7G 125. Similar mechanisms were demonstrated in breast cancers and bladder cancers 127,132. However, it was reported in LUAD and cutaneous melanoma that neutrophils were less infiltrated in group of high m7G levels 56,133. Except for neutrophils, further studies are needed in eosinophils and basophils, which are members of granulocytes as well.

### Others

Nature killer (NK) cells, dendritic cells (DCs) and mast cells belong to innate immune cells which participate in TIME formation. Among them, NK cells serve as the primary defense against cancer, effectively targeting tumor cells without the need for prior sensitization 134. Elevated NK cell abundance is observed among low-risk patients exhibiting favorable prognostic outcomes, typically linked to reduced m7G modifications 115,116,128. DCs function as antigen-presenting cells (APCs), playing a pivotal role in identifying tumor cells and presenting antigens to naïve T cells 135,136. In HCC, patients cluster with m7G levels upregulation suggested immunosuppressive phenotypes and poor prognosis. Meanwhile, these patients showed increased infiltration of DCs as well as decreased activated T cells 115. However, CRC, LUAD and cutaneous melanoma showed opposite results that m7G modification in high-risk patients could suppress DCs infiltrating 56,128,133. These findings align with the double-edged functions of DCs in TIME. Furthermore, mast cells can also accumulate into TIME and exert both pro- and antitumorigenic functions 137. A risk model based on m7G regulated genes in LUAD suggested the connection of advanced stages tumor and mast cells infiltration, and studies indicated different recruitment in different types of cancers 107,108,138.

## M7G regulate TIME by affecting tumor and stromal cells

Except for various types of immune cells, tumor cells and stromal cells are also important components of TIME 139. Investigating the intricacies of immune cell biology governed by m7G modification, scholars have dedicated comparable attention to scrutinizing the impact of m7G within tumor cells and stromal cells. Recent research indicates that m7G modification exerts its influence on both the tumor and stromal constituents of TIME through distinct mechanisms. These mechanisms encompass alterations in the expression of immune checkpoints, secretions of related bioactive factors, as well as the function and communication of stromal cells. A detailed exposition of these aspects is provided below.

### M7G modification impact the immune checkpoints of tumor cells

Immune checkpoints are cell surface molecules of T cells, APCs and tumor cells, which play pivotal roles in modulating immune response 140,141. Based on their diverse functions in T cell activation, immune checkpoints can be categorized into co-stimulatory molecules such as CD28, CD80/CD86, and co-inhibitory ones like PD-1/PD-L1 and CTLA-4^142^. The co-inhibitory immune checkpoints function to diminish the immunogenicity of tumor cells, facilitating their evasion from immune surveillance, which are regarded as crucial immunotherapy targets 143. Specifically, PD-1 and CTLA-4 are predominantly located on the surface of activated T cells, while PD-L1 is expressed on tumor cells in TIME 144. Previous studies of risk models across various cancers indicated that m7G regulators could impact the expression of several immune checkpoints, particularly within tumor cells. In a prognostic risk model of LUAD based on m7G regulators, it suggested that PD-L1 expression was obviously upregulated in high-risk group of patients. Meanwhile, PD-1 and CTLA-4 were overexpressed as well 133,138. Analogous studies in GC, CRC and lower-grade glioma (LGG) showed the overexpression of PD-L1 in patients with high m7G level 105,145,146, and the other immune checkpoint of tumor cells, CD276 showed same outcomes 147. Consequently, m7G modification emerged as a predictive factor for the immunological response to anti-PD-L1 therapy 116,146. These findings underscored the influence of m7G modification on the expression of immune checkpoints in tumor cells, positioning it as a potential biomarker or therapeutic target for immunotherapy.

### M7G modification regulate the secretion of immune related bioactive factors

Except for immune checkpoints located on the surfaces of tumor and immune cells, paracrine signaling through secretion of cytokines, chemokines, IFNs or other immune related bioactive factors is critical for immune response and formation of TIME 148. In recent years, an increasing number of researches have focused on the role of m7G modification in regulating these factors and the downstream signaling pathways. The m7G writer METTL1 was reported to participate in various processes. For example, polyribosome mRNA-seq in ICC cells showed that knockout of METTL1 could reduce translation ratio of the chemokine CXCL8 by affecting the m7G-decoded codon frequency, thereby inhibit the migration of MDSCs to tumors 112. Likewise, researchers also verified the translational activation of TGF-β2 mediated by METTL1-related tRNA m7G modification in pathway analysis of HCC cells 76. And the secretion of TGF-β2 could lead to accumulation of PMN-MDSCs which shaped suppressive TIME and fostered tumor immune evasion 149. Moreover, several pro- and anti-inflammatory cytokines were involved in m7G modification as well. Cytokine composition analysis in prostate cancer cells indicated that the loss of METTL1 could facilitate the secretion of IFN, tumor necrosis factor α (TNF-α) and granulocyte-macrophage colony-stimulating factor (GM-CSF), while downregulate release of macrophage colony stimulating factor (M-CSF), IL10 and IL13^123^. In conclusion, m7G modification played crucial roles in translation and secretion of numerous immune related factors, as well as the regulation of downstream signaling pathways. These processes could also influence the intercellular communication in TIME.

### M7G modification influence the function and communication of stromal cells

Stromal cells in TIME consist of blood and lymphatic endothelial cells, mesenchymal stem cells (MSCs), cancer-associated fibroblasts (CAFs), pericytes and neurons 150. These entities, together with the extracellular matrix (ECM) make up the stromal component. Analogous to immune cells, the function and phenotype of stromal cells have been reported to be influenced by m7G modification and related regulators. In the risk models based on m7G-related genes, researchers utilized the ESTIMATE algorithm to assess stromal scores, thereby evaluating the function of stromal cells in TIME. The results showed higher stromal scores in high-risk subgroups of patients with HCC, GC and ESCC, while SKM and LUAD patients represented opposite outcomes 104,116,145,151,152. These findings indicated diverse activation and accumulation of stromal cells in diverse cancers. Moreover, single cells RNA-seq analysis in HNSCC also revealed that METTL1 knockout could impair direct communication between stromal cells and epithelial cells via inhibition of IL-1b and its receptors 99. Specific to each subtype, the infiltration of CAFs was demonstrated to be positively correlated with the m7G regulator KIF2C, and eIF4E could promote the oncogenic transformation of fibroblasts by facilitating nuclear export of cyclin D1 mRNAs 71,114. Previous studies revealed that the endothelial cell line HUVEC could be shaped by METTL1. Mechanistically, METTL1 could induce the m7G modification of VEGFA mRNA, thus promotes HUVECs migration and angiogenesis 153. However, further investigations are warranted to explore the interaction between m7G modification and other subtypes of stromal cells.

## M7G modification influence the efficacy of adjuvant therapy

Surgical resection remains the primary therapeutic approach for the majority of solid tumors among the various treatment modalities available for cancer management. In recent years, significant advancements in our understanding of tumor biology have paved the way for the integration of adjuvant therapies, including radiotherapy, chemotherapy, immunotherapy, and combination therapy. These adjunctive treatments aim to reduce tumor burden, downgrade primary tumor staging, and increase the feasibility of surgical intervention 154. However, the effectiveness of adjuvant therapies remains constrained by factors such as drug susceptibility, tolerance, and the potential for tumor recurrence. Building upon prior research, it has become evident that m7G modification plays a pivotal role in modulating the outcomes of various adjuvant therapies across different cancer types. These findings are comprehensively discussed in the following sections and summarized in Table 2.

### Immune checkpoint blockade (ICB) therapy

As ICB therapy has demonstrated substantial efficacy and promise in cancer treatment over the past several decades, immune checkpoint inhibitors (ICIs) have found application in clinical practice for attenuating the inhibitory signals hindering T-cell activation and promoting anti-tumor immune responses 155. In contemporary clinical practice, ICIs predominantly encompass agents targeting CTLA-4, PD-1, and PD-L1. The pioneering success in cancer immunotherapy was achieved with the anti-CTLA-4 antibody, Ipilimumab. Rigorous double-blind phase II trials conducted in advanced melanoma demonstrated that Ipilimumab monotherapy significantly prolonged overall survival and exhibited a dose-dependent effect on antitumor efficacy 156. Recent investigations have uncovered the therapeutic potential of Ipilimumab not only in monotherapy but also in combination with other ICIs or chemotherapeutics 157-159. Anti-PD-1 antibodies are ICIs with most variety of approval oncology therapeutic products in clinic application, such as Pembrolizumab and Nivolumab 160. Both monotherapy and combination based on these inhibitors showed prognostic benefit in patients of various cancers, and the treatment effect was enhanced with overexpression of PD-L1^161^-^163^. In order to improve the efficacy and safety of ICB therapy, researchers nowadays have paid more attention to bispecific antibodies which simultaneously target 2 different immune checkpoints, and there have been numerous associated phase I or phase II clinical trials in types of cancers 164-166. Apart from PD-L1 expression, tumor mutation burden (TMB) and tumor immune dysfunction and exclusion (TIDE) were used to assess the treatment response in the prevailing views. TMB represent the neoantigens generated by the mutations in tumor, which can induce T cells recognition and immune response 167. TIDE reflect the induction of T cells dysfunction and prevention of CTLs infiltration which lead to immune evasion in TIME 168. Generally, higher TMB and lower TIDE may predict enhanced efficacy of ICB therapy. Previous evidence suggested that m7G modification and its regulators could influence the response to ICB therapy in many cancers. On the one hand, m7G scores based on m7G levels and prognostic risks were important biomarker in ICB therapy. Clustering analyses conducted in GC, CRC, LUAD and bladder cancers revealed a noteworthy association between higher m7G risk scores and lower TMB. This correlation was further linked to diminished responses to ICB therapy and a poorer prognosis for patients. Additionally, TIDE scores demonstrated a consistent negative correlation with m7G levels across various cancer types 105,109,138,169. These observations underscored the potential of m7G as a valuable biomarker for predicting responses to ICB treatment. For the concrete ICB therapy strategies, researchers examined the predictive value of m7G signature in different immunotherapy cohorts. According to the anti-PD-1 and anti-PD-L1 immunotherapy cohorts, low-risk group of patients in HCC, SKM, LGG and bladder cancers showed significant clinical benefits for anti-PD-1 or anti-PD-L1 treatment 115,116,146,170. However, the scenario is nuanced when considering combination therapy. In PDAC, the efficacy of anti-PD1 and anti-CTLA-4 combined treatment in high-risk group patients was better than low-risk group, while single ICI showed opposite outcomes 125. These findings underscored that ICB therapy efficacy was negative correlated with risk scores based on m7G levels, while the regulation in combination therapy was still complicated.

On the other hand, some specific m7G-related genes, especially m7G writers play important roles in ICB therapy. METTL1 stands as the most extensively studied m7G methyltransferase within the context of tumor immunity. In ICC, researchers constructed hydrodynamic transfection models of METTL1 knockout mice. The results suggested that METTL1 knockout could increase the proliferation and antitumor activity of T cells, as well as boost the efficacy of anti-PD-1^112^. More recently, investigations in prostate cancer indicated that knockout of METTL1 could enhance the sensitivity of ICB therapies containing anti-PD1 and anti-CTLA-4 antibodies 123. Furthermore, a comprehensive analysis of pan-cancers also explored whether METTL1 could influence the efficacy of anti-PD-L1 therapy. The expression of PD-L1 was in negative proportion to METTL1, but patients with higher METTL1 expression showed more responses to anti-PD-L1 therapy. These findings revealed that METTL1 may not be suitable target of combination therapy with anti-PD-L1^97^. In conclusion, m7G modification as well as the related regulators are important biomarkers and potential targets in ICB therapy, and investigation of METTL1 inhibitors may provide innovative strategies to enhance the efficacy of ICIs like anti-PD1 and anti-CTLA-4.

### Chemotherapy

Chemotherapy is a cornerstone treatment modality across a wide spectrum of cancer types, and resistance to chemotherapy stands as the foremost determinant of unfavorable prognoses. Notably, M7G modification has emerged as a factor with significant influence on the resistance of traditional chemotherapy in various cancers. For example, in NPC, m7G modification of tRNAs catalyzed by METTL1/WDR4 complex could accelerate codon recognition and mRNA translation, which contributed to activation of WNT/β-catenin pathway and subsequently promoted chemoresistance of NPC cells to cisplatin and docetaxel 73. Similarly, m7G tRNA modification in OS heightens chemoresistance to doxorubicin by increasing mRNA translation 171. Except for m7G writers, some m7G readers including EIF4E and QKIs have also been implicated in the regulation of chemoresistance. The classical antiviral drug Ribavirin is a mimic of m7G-caps, which can inhibit nuclear export of EIF4E. Researchers applied Ribavirin in M4/M5 AML patients and showed its clinical benefits 172. Boussemart et al. uncovered that EIF4E cap-binding protein could form a complex with EIF4G and EIF4A, thereby suppressing the therapeutic efficacy of anti-BRAF and anti-MEK compounds in melanoma, colon, and thyroid cancer cell lines. Subsequent investigations unveiled that the inhibition of EIF4E-EIF4G interaction and translation activation could overcome the chemoresistance of these chemotherapy drugs 173. Moreover, QKI7-mediated internal mRNA m7G modification were involved in chemoresistance upon doxorubicin. The overexpression of QKI7 in HeLa and HCC cells could enhance doxorubicin-induced apoptosis and sensitized cancer cells to chemotherapy drugs 52. These findings underscored diverse functions of m7G regulators in tumor initiation and progression as well.

Apart from the traditional chemotherapy drugs, previous studies indicated that sensibility and resistance of molecular targeted therapies could also be regulated by m7G modification. For instance, the tyrosine kinase inhibitor (TKI) Lenvatinib is the first-line therapy for HCC, and the Lenvatinib-resistant patients showed increased level of m7G modification 174. Further studies demonstrated that the resistance of Lenvatinib were enhanced by translational activation of EGFR, which was dependent on tRNA m7G modification catalyzed by METTL1/WDR4 complex 175. Likewise, the other TKI in HCC therapy, Sorafenib was reported to be associated with WDR4. WDR4 induced translation of CCNB1 mRNA by tRNA m7G modification, thus promoted sorafenib resistance 74. Furthermore, it is imperative to undertake further investigations to unravel the intricate interplay between targeted therapies and m7G modification in various other cancer types.

### Radiotherapy and radiofrequency ablation (RFA)

Radiotherapy and RFA belong to local treatments of cancers. Notably, radiotherapy has gained prominence as an essential therapeutic approach in the management of HCC. The efficacy of radiotherapy is based on DNA damage, thus the radioresistance of cancer cells are associated with DNA DSB repair. Liao et al. demonstrated that METTL1 could render HCC cells resistant to IR by upregulating DNA-PKcs and DNA ligase IV translation and promoting non-homologous end-joining (NHEJ) mediated repair 176. RFA is an effective local therapeutic strategy of various cancers, and the post-RFA tumor recurrence is the dominant factor to influence the therapeutic effects. Investigation of RFA and m7G modification mainly concentrated on HCC. Researchers have demonstrated that insufficient RFA could upregulate METTL1 and lead to immunosuppressive TME, which resulted in PMN- MDSCs and residual HCC progression 76. Furthermore, further studies by the same team indicated that METTL1-mediated tRNA m7G modification could facilitate post-RFA HCC metastasis by inducing translation of SNAI1 and SLUG, which provide potential therapy target to decrease recurrence rate 177. To achieve a comprehensive understanding of m7G modification in conjunction with RFA, it is imperative to conduct further research encompassing various cancer types in the future.

## Conclusion and prospects

In this comprehensive review, we mainly focused on the m7G modification and its pivotal role in the regulation of the TIME and adjuvant therapies. The m7G modification, a prevalent RNA modification in eukaryotes, has garnered substantial attention from researchers in recent decades. This heightened interest can be attributed to the advent of advanced methodologies for the detection of m7G, including LC-MS/MS, m7G-seq, m7G-MeRIP-seq and m7G-miCLIP-seq. These innovative approaches have shed light on the pervasive nature of m7G modification, implicating its involvement not only in mRNAs but also in numerous ncRNAs, including tRNAs and rRNAs. To date, clearly reported regulators of m7G modification contain the m7G writer complexes such as RNMT/RAM, METTL1/WDR4 and WBSCR22/TRMT122, the m7G-caps readers CBC and EIF4E, as well as internal m7G binding protein QKI. Despite some m7G-related genes and bridge genes were reported in many integrated analyses and prognostic models, their specific mechanisms of regulating m7G modification were elusive without concrete supporting evidence. Apart from these m7G regulators, some other internal and external regulation factors including aberrant epigenetic modification, drug or IR treatments, as well as some oncogenic molecules and signaling pathways are also involved in tumor initiation and progression.

**Figure 4:**
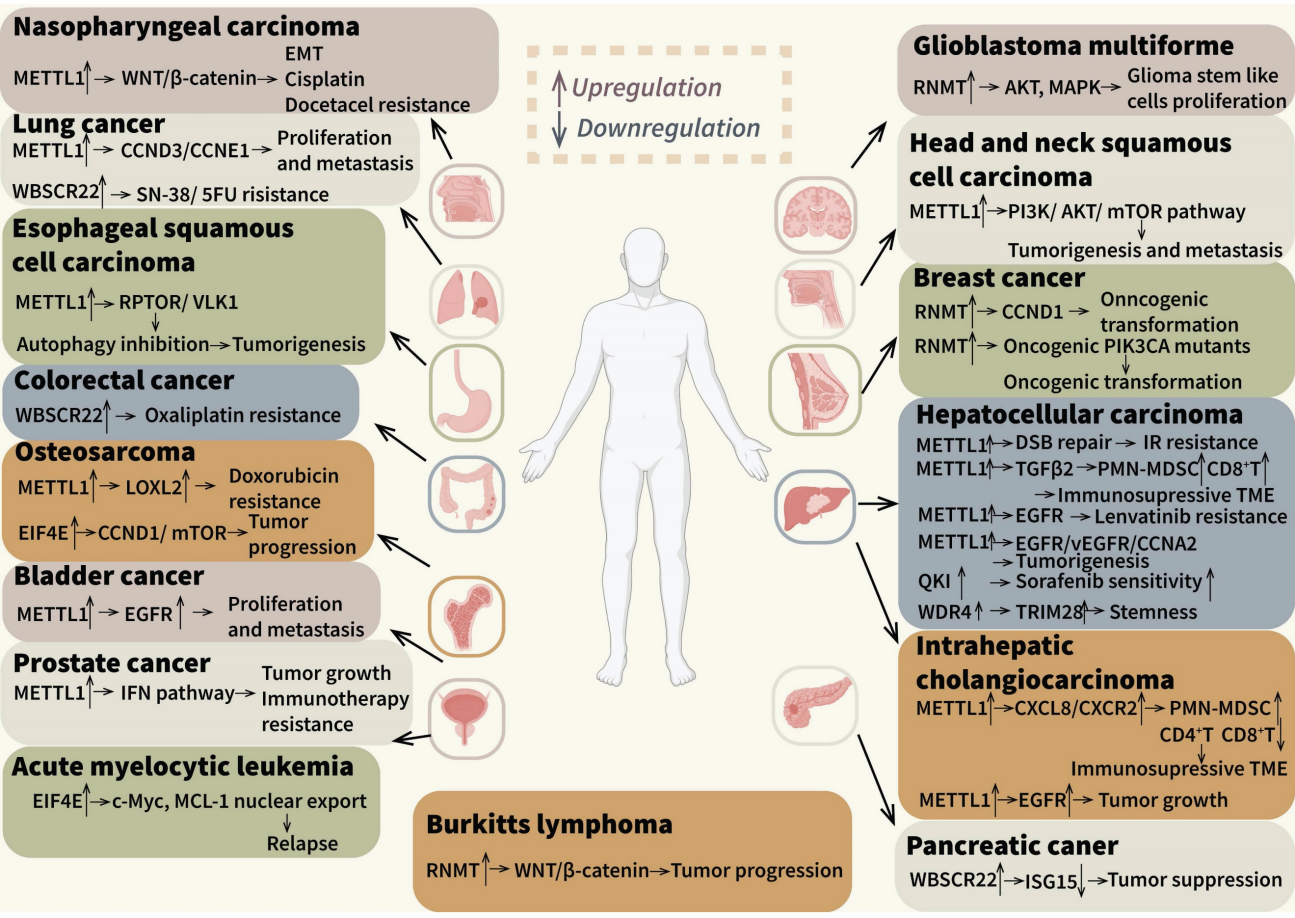
** The role of m7G modification and its regulators in diverse cancers.** M7G modification and its regulators have been reported in many types of cancers. The related cancers and downstream genes and pathways are showed in the figure.

We have systematically reviewed the roles of m7G modification in regulating tumor initiation and progression of different malignancies, including various solid tumors, leukemia and lymphoma, as it illustrated in Figure 4. (Figure 4). Firstly, distinct cancer-associated m7G mechanisms manifest in different cancer types. Dysregulation of m7G-caps modification emerges as a primary mechanism associated with tumor progression and relapse of breast cancers and hematological malignancies, while aberrant internal m7G is more prevalent in HCC and ICC 48,172,178. Secondly, diverse downstream pathways like EGFR/MAPK, AKT/mTOR and WNT/β-catenin were involved in different cancer types, which might cause different pathological behaviors 73,99,175. Furthermore, we observed varied effects on tumor treatment outcomes. It was disclosed that m7G modification could mediate IR resistance and relapse after RFA, but researches in other tumors mainly focused on chemotherapy like platinum drugs. These findings were also reported in previously published reviews. Notably, the regulation of immune cell biology within the TIME was implicated. Herein, we concentrated on the risk models based on m7G-related genes or different expression genes and some mechanistic researches. The infiltration and recruitment of immune cells including T cells, B cells, MDSCs, macrophages and neutrophils were correlated with m7G modification levels and regulators, which simultaneously showed different outcomes in distinct types of cancers. In addition, the stromal cells and tumor cells in TIME can also be regulated by m7G modification, which can influence immune checkpoints expression, cytokines and chemokines secretion, as well as the functions of stromal cells. Furthermore, researchers have suggested that m7G modification is associated with the efficacy of many adjuvant therapies. In ICB therapies, m7G-related risk scores were negative correlated to curative activity of ICIs, and the m7G writer METTL1 could inhibit efficacy of ICB therapies. Nevertheless, challenges persist in terms of efficacy and safety in the monotherapy of ICIs, necessitating the exploration of combination therapies in various cancers 179. Mechanistically, chemotherapy drugs and radiation can activate immunostimulatory signals via eliminating the tumor cells, thereby promoting the cytotoxicity of immune effector cells and suppressing the formation of suppressive TIME 180. These observations underscored the imperative for further investigations into alternative anticancer treatments. Previous studies demonstrated that chemotherapy, radiotherapy and RFA could also be influenced by m7G regulators METTL1, EIF4E and QKI7. Apart from the solid tumors, some hematologic tumors like AML also involved in m7G-related combination therapies.

Previous investigations have demonstrated the significance of m7G and its regulators as important biomarkers for diagnosis, prognosis and immunotherapy in various cancers. Overall, m7G levels are positively correlated with the TMN stages in most cancers, and high m7G scores in patients usually predict worse prognosis. Moreover, m7G levels may affect the TMB and TIDE scores, as well as the expression of immune checkpoints, thereby influence the efficacy of immunotherapy 75. In terms of m7G regulators, METTL1 and WDR4 have been regarded as pivotal oncogenic molecules associated with poor overall studies, which also provide possibilities for early diagnosis and treatment 14,51. Simultaneously, other regulators and m7G-related genes have showed the expression and functions in various cancers, with only a few members showing downregulation in tumor tissues and association with clinical benefit. These findings have also revealed the oncogenic role of m7G in most of cancers. However, relevant clinical trials targeting m7G and m7G regulators are still lacking, and most of researches to date are based on mechanistic exploration and clustering analysis. From another perspective, the research status indicates great potential for targeting m7G modification in clinic application, especially combination therapies. For example, knocking down METTL1 by using inhibitors, antibodies or nanomedicine methods can be served as promising therapy strategies, as mechanistic studies have illustrated that the efficacy of ICIs and certain chemotherapeutic agents is augmented in METTL1 knockout mice. This suggests a synergistic effect when combining these therapeutics with m7G-targeted therapy. As targeted therapy continues to advance, novel inhibitors of m7G regulators may find application in adjuvant therapy alongside ICIs and other targeted drugs. In summary, m7G modification emerges as a pivotal factor in regulating the TIME and enhancing adjuvant therapy efficacy. Consequently, it holds promise as both a potential biomarker and a target for combination therapy.

## Figures and Tables

**Table 1:** Biological functions and mechanisms of m7G modification in tumor-infiltrating immune cells

Immune cells		Cancer types	Related m7G regulators	Functions and mechanisms	Ref.
T cells	CD4^+^ cells	HCC	METTL1	More infiltration of Tregs and activated memory T cells by METTL1 overexpression	97
		LUAD	/	More infiltration of Tregs and Th cells in low-risk patients	104
		Breast cancers	METTL1	More infiltration of Tregs by METTL1 overexpression	97
			/	More infiltration of Tregs and Th cells in high-risk patients	132
		GC	/	More infiltration of Th cells in low-risk patients	88
		HNSCC	METTL1	Decreased infiltration of Tregs in METTL1-KO mice	99
	CD8^+^ cells	LUAD	WBSCR22	More infiltration of activated CD8^+^ cells	101
		GC, prostate cancers, STS	/	More infiltration of CD8^+^ cells in low-risk patients	86,88,89
		HNSCC	EIF4E3, NCBP2	More infiltration of CD8^+^ cells in low-risk patients	85
B cells	Naïve B cells	CRC, LUAD	/	More infiltration of naïve B cells in low-risk patients	104,105
		Cutaneous melanoma	/	More infiltration of naïve B cells in high-risk patients	106
	Memory B cells	GC, HCC, bladder cancer	/	More infiltration of memory B cells in low-risk patients	107-109
	Plasma cells	GC, HCC	/	More infiltration of plasma cells in low-risk patients	108,109
MDSCs		ICC	METTL1	More CXCL8 translation and consequent PMN-MDSC infiltration regulated by METTL1	112
		HCC	METTL1	Activated TGF-β pathway and more PMN-MDSC infiltration regulated by METTL1	76
			NUDT16	More infiltration of MDSCs in high-risk patients	114,115
		SKM	EIF4E3, IFIT5	More infiltration of MDSCs in low-risk patients	116
Macrophages		Prostate cancer	METTL1	Suppressed IFN signaling pathway and M2 macrophages polarization mediated by METTL1	123
		LUAD	WDR4	Enhanced expression of CD73 and infiltration of CD206^+^ M2 macrophages	98
		CRC, ovarian cancer	/	More infiltration of M1 macrophages in low-risk patients	96,126
		Bladder cancer	/	More infiltration of M1 macrophages in high-risk patients	127
		GC, PDAC	/	More infiltration of M2 macrophages in high-risk patients	109,125
Monocytes		CRC, bladder cancer	/	More infiltration of monocytes in low-risk patients	127,128
Neutrophils		PDAC	FN1, ITGB1	More infiltration of neutrophils in high-risk patients	125
DC		HCC	NUDT16	More infiltration of DCs in high-risk patients	115
		CRC, LUAD	/	More infiltration of DCs in low-risk patients	128,133
NK cells		CRC, SKM, LUAD	/	More infiltration of NK cells in low-risk patients	116,128,138

M7G modification and its regulators exert important functions in immune cell biology of TIME. The infiltration of T cells, B cells, MDSCs, macrophages, monocytes, DCs and NK cells has been reported to be influenced by m7G modification, and several different types of cancers are involved.

**Table 2:** The roles of m7G modification in adjuvant therapies of cancers

Therapeutic methods		Cancer types	Related m7G regulators	Functions and mechanisms	Ref.
ICB therapy	Anti-PD-L1, anti-PD-1, anti-CTLA-4	HCC, PDAC, SKM, GC, LGG, etc.	/	Increased treatment responses in patients of low-risk groups	109,115,116,125
	Two-drugs combination therapy	PDAC	/	Better efficacy in patients of high-risk groups	125
	Anti-PD-1	ICC	METTL1	Enhanced efficacy by co-blockade of METTL1 and downstream chemokine pathways	112
	Anti-PD-1, anti-CTLA-4	Prostate cancer	METTL1	Enhanced efficacy by METTL1 inhibition	123
Chemotherapy	Cisplatin and docetaxel	NPC	METTL1/WDR4	Promoted chemoresistance by METTL1 and WNT/β-catenin pathway	73
	Doxorubicin	OS	METTL1/WDR4	Promoted chemoresistance by METTL1	171
		HCC, cervical cancer	QKI7	Promoted chemosensitivity by QKI7-mediated inhibition of Hippo pathways	52
	Anti-BRAF and anti-MEK compounds	Melanoma, CRC, thyroid cancer	EIF4E	Decreased resistance by combination therapy with inhibition of EIF4E-EIF4G interaction	173
	Ribavirin	AML	EIF4E	Clinical benefits of m7G-cap mimic Ribavirin by targeting EIF4E	172
	Lenvatinib	HCC	WDR4	Promoted chemoresistance by WDR4 and TRIM28	174
		HCC	METTL1	Promoted chemoresistance by METTL1 and EGFR pathway	175
	Sorafenib	HCC	WDR4	Promoted chemoresistance by MYC-targeted WDR4 and CCNB1	74
Radiotherapy		HCC	METTL1	Promoted IR resistance by METTL1-mediated NHEJ-based DNA repair	176
RFA		HCC	METTL1	Immunosuppressive TME and residual HCC progression by upregulation of METTL1 in post-RFA recurrent tumor	76
		HCC	METTL1	Increased post-RFA HCC metastasis by METTL1-mediated upregulation of SNAI1/SLUG axis	177

m7G modification plays a pivotal role in modulating the effectiveness of various adjuvant therapies, including ICB therapy, chemotherapy, radiotherapy and RFA. The different cancer types and concrete mechanisms are shown above.

## Abbreviations

M7G: N7-methylguanosine
m6A: N6-methyladenosine
mRNA: messenger RNA
tRNA: transfer RNA
rRNA: ribosomal RNA
ncRNA: non-coding RNA
miRNA: microRNA
snoRNA: small nucleolar RNA
RNMT: RNA guanine-7 methyltransferase
RAM: RNMT-activating miniprotein
METTL1: methyltransferase-like 1
WDR4: WD repeat domain 4
WBSCR22: Williams-Beuren syndrome chromosomal region 22
TRMT112: tRNA methyltransferase activator subunit 11-2
CBC: cap-binding complex
EIF4E: eukaryotic translation initiation factor 4E
TIME: Tumor immune microenvironment
MDSC: myeloid-derived suppressor cell
TAM: tumor-associated macrophages
DC: dendritic cell
ICB: immune checkpoint blockade
ICI: immune checkpoint inhibitors
TKI: tyrosine kinase inhibitor
HCC: hepatocellular carcinoma
ICC: intrahepatic cholangiocarcinoma
GC: gastric cancer
CRC: colorectal cancer
LUAD: lung adenocarcinoma
PDAC: pancreatic ductal adenocarcinoma
HNSCC: head and neck squamous cell carcinoma
AML: acute myelocytic leukemia
